# Hot Fingers: Individually Addressable Graphene-Heater Actuated Liquid Crystal Grippers

**DOI:** 10.1021/acsami.4c06130

**Published:** 2024-06-13

**Authors:** Laura S. van Hazendonk, Zafeiris J. Khalil, Wilko van Grondelle, Levina E. A. Wijkhuijs, Ingeborg Schreur-Piet, Michael G. Debije, Heiner Friedrich

**Affiliations:** † Laboratory of Physical Chemistry, Department of Chemical Engineering and Chemistry Eindhoven University of Technology, P.O. box 513, Eindhoven 5600 MB, The Netherlands; ‡ Institute for Complex Molecular Systems, Eindhoven University of Technology, P.O. box 513, Eindhoven 5600 MB, The Netherlands; § Center for Multiscale Electron Microscopy, Department of Chemical Engineering and Chemistry, Eindhoven University of Technology, P.O. box 513, Eindhoven 5600 MB, The Netherlands; ∥ Stimuli-responsive Functional Materials and Devices (SFD), Department of Chemical Engineering and Chemistry, Eindhoven University of Technology, P.O. box 513, Eindhoven 5600 MB, The Netherlands

**Keywords:** liquid crystal network, actuators, graphene, soft robotics, direct ink writing

## Abstract

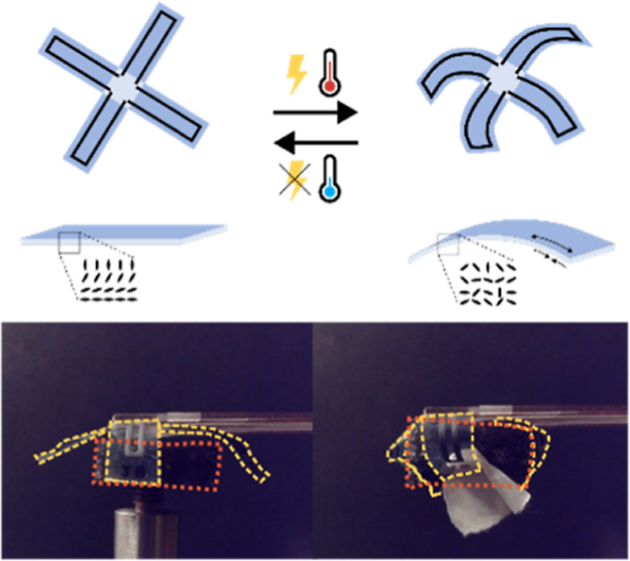

Liquid crystal-based actuators are receiving increased attention for their applications in wearables and biomedical or surgical devices, with selective actuation of individual parts/fingers still being in its infancy. This work presents the design and realization of two gripper devices with four individually addressable liquid-crystal network (LCN) actuators thermally driven via printed graphene-based heating elements. The resistive heat causes the all-organic actuator to bend due to anisotropic volume expansions of the splay-aligned sample. A heat transfer model that includes all relevant interfaces is presented and verified via thermal imaging, which provides good estimates of dimensions, power production, and resistance required to reach the desired temperature for actuation while maintaining safe electrical potentials. The LCN films displace up to 11 mm with a bending force of 1.10 mN upon application of 0–15 V potentials. The robustness of the LCN finger is confirmed by repetitive on/off switching for 500 cycles. Actuators are assembled into two prototypes able to grip and lift objects of small weights (70–100 mg) and perform complex actions by individually controlling one of the device’s fingers to grip an additional object. Selective actuation of parts in soft robotic devices will enable more complex motions and actions to be performed.

## 1 Introduction

Stimuli-responsive materials have been receiving increased attention, especially for motile devices, biomedical applications including wearables and surgical devices, dynamic surfaces, and materials for personal comfort.^1−4^ Liquid crystal (LC) actuators based on LC elastomers (LCEs) or LC networks (LCNs) are prime candidate materials for use in soft-touch devices^2,5−8^ and are promising alternatives to, for example, hydrogels^9^ and silicones.^10^ LCE/LCN grippers have structures which can bend due to internal disruption of the LC mesogenic order with large deflections at relatively low actuation temperatures, facilitating soft gripping without introducing damage to the object.^5,11−13^ Nevertheless, soft actuators with control over individual gripper fingers are still in their infancy and often are not optimized: for example, they cannot independently actuate their fingers^14−18^ or are overly complex in design.^19,20^

There is a clear need for each of the gripper’s fingers to be individually actuated upon exposure to a stimulus (stimuli options include light,^21^ electrical^22^ or magnetic^23^ fields, piezoelectricity,^24−26^ and humidity^27^) to realize complex gripping actions comparable, for example, to a human hand. Local heating appears a viable option for actuation but is currently primarily achieved through the incorporation of metal wires,^18^ kapton films,^28^ submersion in water baths,^29^ placing the actuator on a hot plate,^30^ heating the surroundings,^30,31^ thermal emissions by the object to be grasped,^32^ or by locally irradiating with light.^33^ In contrast, 3D printing of resistive heaters directly on top of the LCN actuators would dramatically expand the design freedom, and actuation modes could potentially be changed by simply varying the print pattern. Suitable printed flexible circuits have been produced from conductive inks based on metals,^34^ conductive polymers,^35,36^ or carbon complexes.^37−39^ While most heat-actuated LCN soft robots have been actuated via metals, noncorrosive resistive heating elements like graphene tolerate more humid and saline environments, especially relevant in medical applications. In addition, graphene is abundant, skin compatible, and both thermally and electrically conductive, making it a promising candidate for printed electronics,^40^ especially for wearables,^41,42^ supercapacitors,^43^ and biomedical sensors.^44^ Graphene-based inks can be deposited onto substrates by additive manufacturing approaches including 3D printing by using direct ink writing (DIW)^39,45^ or screen-printing techniques.^42^ However, the coupling of graphene-based resistive heaters with LC-based materials to form all-organic actuators has not yet been shown.

Here, we design and build gripper devices by printing graphene tracks that function as local resistive heating elements on splay-aligned LCN actuators. A heat transfer model that includes all relevant interfaces is established and verified to aid device design linking heater and LCN dimensions, applied voltage, and achievable actuation temperatures. Performance tests demonstrate robust, reversible actuators that can be operated at safe potentials below 15 V. Initial insights into the bending force produced by the LCNs are also established. Finally, two gripper prototypes, each having four fingers, are presented, demonstrating the ability to grip and lift objects and perform complex gripping actions by individually addressing the device’s fingers.

## 2 Results and Discussion

**Figure 1:**
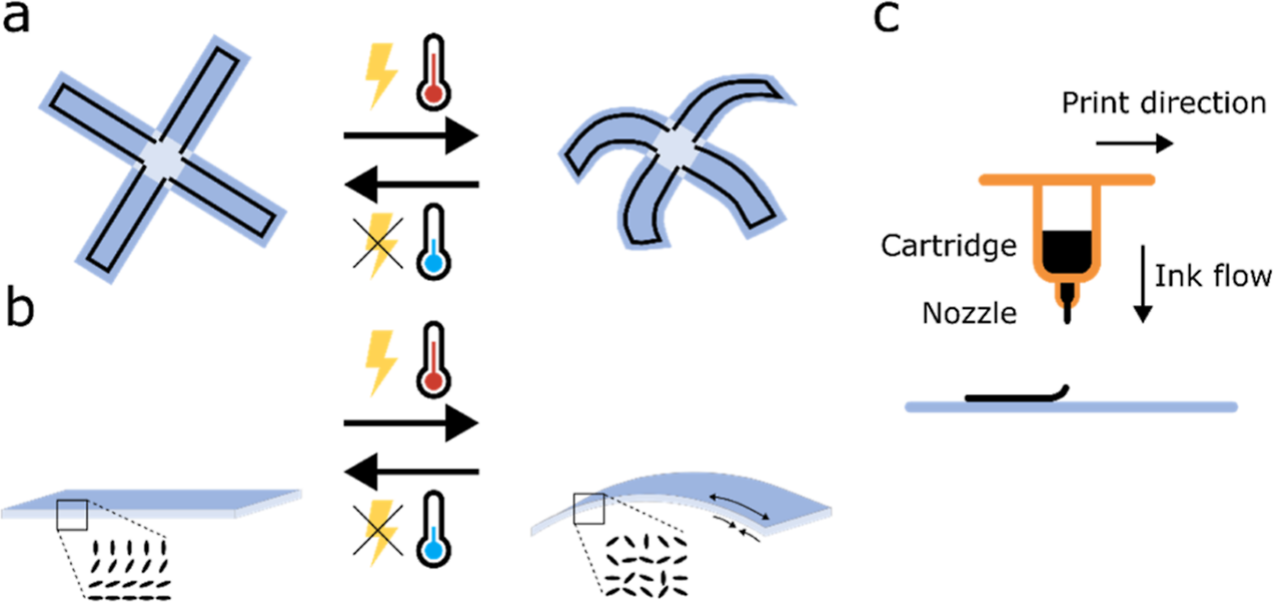
a) Schematic of the basic structure and function of the actuator fingers formed by the LCN (blue) and printed graphene tracks (black). When addressed by an electrical potential, the graphene tracks resistively heat the LCN, prompting thermal disruption of the splay-aligned LCN and hence inducing bending of the actuator fingers. Upon switching off the current flow, the heat dissipates and the actuators return to their initial flat states. (b) Schematic of the macroscopic and mesoscopic reversible deformation of a splay-aligned LCN with the planar side on the inside of the curvature: the black ellipsoids schematically represent the monomers in the splay-aligned LCN morphology (left), which gets disrupted upon heating (right), leading to expansion of the film on one face and contraction on the other (thin arrows), resulting in macroscopic bending. (c) Schematic showing DIW of the graphene ink using a printer consisting of an ink cartridge with a nozzle through which the ink is extruded on the substrate.

A schematic of a gripping device consisting of four “fingers” and its actuation is shown in Figure 1a. LCNs were chosen as the stimuli-responsive material, as the mesogenic alignment (Figure 1b) can be easily controlled by using alignment cells. LCNs with splay alignment generally produce greater bending than other alignments^46^ and are generally stiffer than LC elastomers (LCEs),^47^ which is expected to result in larger bending forces. In this work, a standard LCN network was formed from common monomers 4-methoxyphenyl-4-(6-acryloyloxyhexyloxy)benzoate (RM105) and 1,4-di(4-(6-acryloyloxyhexyloxy)benzoyloxy)-2-methylbenzene (RM82) and a photoinitiator: bis(2,4,6-trimethylbenzoyl)phenyl phosphine oxide (Irgacure 819) (Figure S1 in the Supporting Information) based on a previously published protocol^46^ (details in the Experimental Section). The LCN network exhibits bending between 40 and 60 °C as the nematic order is disrupted.^46^ Differential scanning calorimetry (DSC), dynamic mechanical thermal analysis (DMTA), and polarized optical microscopy results confirmed structural changes within this temperature range (Figures S2, S3 and S4), and observation of the samples through crossed polarizers confirmed the alignment of the LCN. The DMTA measurements reveal that the LCN storage modulus gradually decreases from 1.3 × 10^3^ MPa at room temperature to 4.1 × 10^2^ MPa at 60 °C and 1.3 × 10^1^ MPa at 100 °C, while the loss tangent peaks at around 67 °C. These observations are consistent with those for similar LCNs in the literature.^48^ To locally increase the temperature, graphene tracks were precisely deposited on the homeotropic side of the splay LCN films by DIW (Figure 1c).

The relation between graphene track length and cross-sectional area, voltage, current, heating capabilities of the graphene tracks and LCN actuator dimensions was modeled to aid in device design. The model provides good estimates of dimensions, power production, and necessary resistance required to reach the desired temperature for actuation while maintaining safe electrical potentials. Electrical current passing through a resistive heater causes its temperature to increase; via thermal transport across the heater/LCN interface, this increase is used to initiate bending in the thermally sensitive LC actuators. Given that the temperature required to generate actuation is determined by the LCN, we aimed to predict the applied voltage range required to reach this temperature for specific LCN and heater configurations, taking thermal losses across the interfaces to the environment into account. To this end, we modeled the heat transfer rates using a heat transfer balance
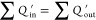
1

The system has only one input, which is the applied electrical power. It was assumed that all electrical power (*P*) is converted into heat by the graphene track (*Q*′_in,*t*_). The graphene track (referenced with a subscript *t* in the model parameters) was assumed to have a conductive heat transfer rate to the substrate (*Q*′_cond,*t*_) and convective (*Q*′_conv,*t*_) and radiative (*Q*′_rad,*t*_) heat transfer rates to the surrounding air
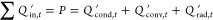
2

**Figure 2:**
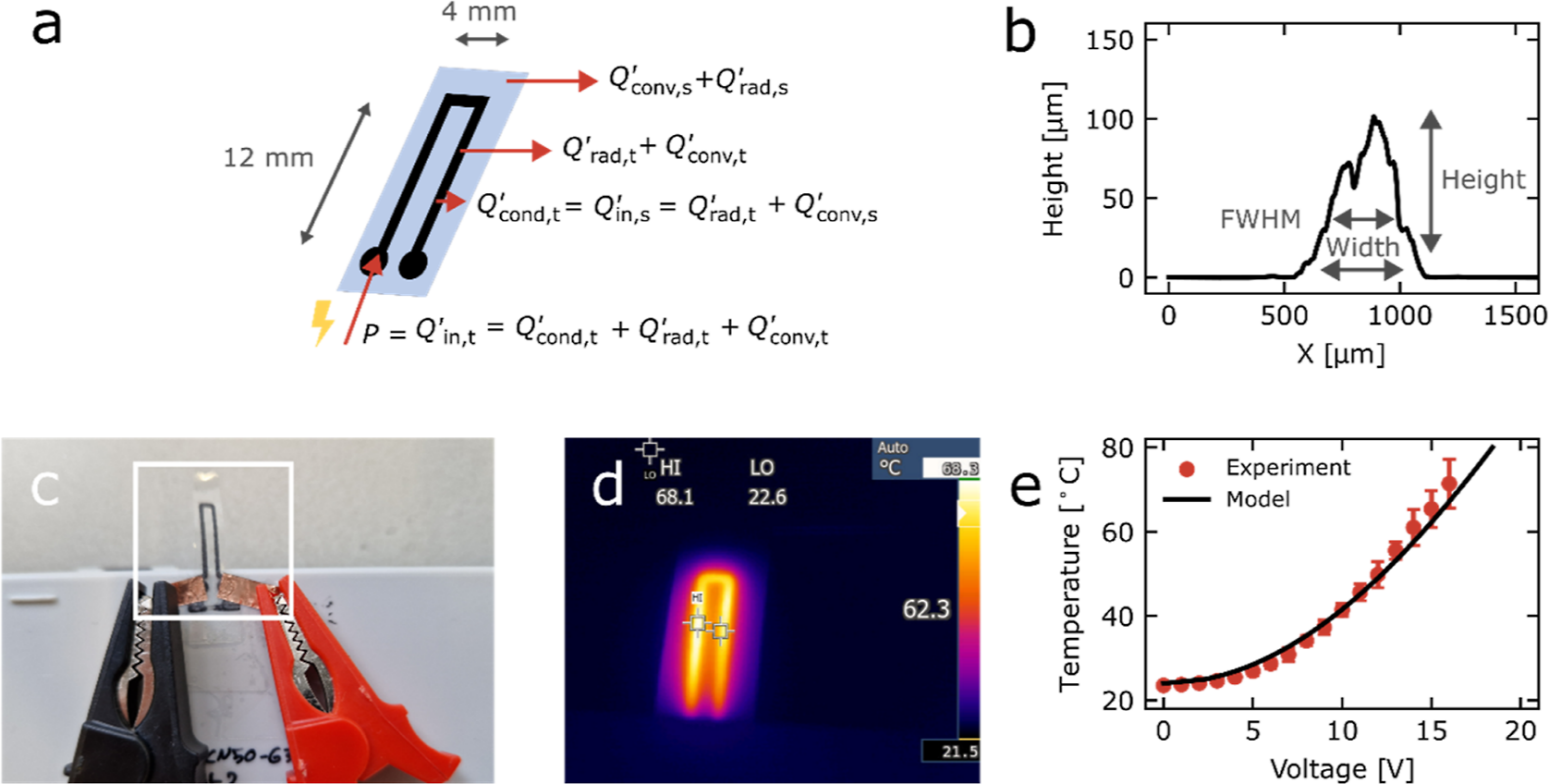
(a) Schematic illustration of the heat transfer rates into and out of a single actuator finger (leg-to-leg distance of 4 mm and a heater length of 12 mm), where the symbols refer to eqs 1–7. (b) Typical profilometer measured cross-section of a printed graphene track with height, width, and full width at half-maximum height (FWHM) defined. (c) Photograph of a single finger taped to a plastic stage and connected to the power unit using crocodile clips and copper tape. (d) Display of the infrared (IR) camera showing a thermal image acquired at 15 V. (e) Temperature versus voltage plot for measured data points for a typical sample (red dots with standard deviations) and as predicted by the model (black curve).

*Q*′_cond,*t*_ was assumed to be the input heat transfer rate to the substrate (referenced with a subscript *s* in the model parameters) heat transfer rate balance (*Q*′_in,s_ = Q’_cond,t_). We assumed the substrate to have convective (*Q*′_conv,*s*_) and radiative (*Q*′_rad,*s*_) heat transfer rates to the surrounding air from both the bottom and top surfaces of the device, while the edges were neglected (see Figure 2a)
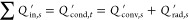
3

By combination of the individual heat transfer rate balances of the graphene track and substrate, a system balance can be written
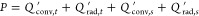
4

The radiative heat transfer rate (*Q*′_rad_) was calculated according to eq 5 where σ is the Stefan–Boltzmann constant, ε_s_ and ε_t_ are the emissivity of the materials (substrate and track), *T*_h_ is the desired temperature of the substrate, *T*_c_ is the temperature of the cold surroundings, and *A*_h,s_ is the surface area of heat transfer of the substrate, which equals the combined top and bottom surface areas of the LC actuator minus the area covered with the printed track. In contrast, *A*_h,t_ is the surface area of the printed track
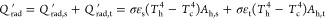
5

The convective heat transfer was calculated using the convective heat transfer coefficient, *H*_L_, for the desired temperature increase Δ*T* = *T*_h_ – *T*_c_
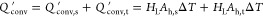
6

The heat transfer coefficient *H*_L_ was calculated according to an empirical approximation in lab conditions found in the literature^49,50^
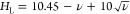
7

In Equation 7, *v* is the airflow [m s^–1^].

From eqs 7 and 1 and the sample resistance (*R*), required input powers (*P*) and thus input voltages (*V*) were estimated
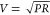
8where some initial test prints (Figure S5) were used to get an estimate of the resistance *R*. For safety reasons, the potential should be as low as possible, ideally below 20 V (ca. 12 mA for our conductors).^51^ Model input parameters are listed in Table S1. The spreadsheet containing the model implementation may be accessed via the 4TU ResearchData repository.^52^ Note that with the presented model, one can scale the actuator fingers while preserving a safe operating voltage by adjusting the cross-sectional area, i.e., the resistance, of the heating element, using the same scaling factor as that of the substrate. Scaling of the bending force is not straightforward and may be approached by optimization of the LCN network and film thickness.

To test if the optimized resistive heaters function as predicted by the model, 35 graphene track samples were printed on LCNs, which were subsequently cut into 15 × 5 mm^2^ strips. The average resistance of the graphene tracks was 1.96 ± 0.37 kΩ, their height 76 ± 17 μm, and FWHM 543 ± 174 μm (the track shape and profilometer measured cross section are shown in Figure 2a,b, respectively, with further details in Table S1 and Figure S6). The heating behaviors of two representative samples were tested by connecting the graphene track to a power unit with copper tape and clamps (Figure 2c). The applied voltage was gradually increased from 0 to 16 V, while the surface temperature was registered by a hand-held infrared (IR) camera (Figure 2d). For more accurate characterization, both ends of the sample were fixed to prevent sample movement, so that the IR camera could easily focus on the sample during heating. At each change in applied potential, the sample was given time to reach a steady temperature. These experiments established that the presented model predicts the achievable temperatures rather well, and potentials of around 14 V are sufficient to achieve the desired 60 °C for this LC actuator (Figure 2e). The model may be easily adapted to other LCNs formed using monomers with lower actuation temperatures^53^ or to actuators with different dimensions.

**Figure 3:**
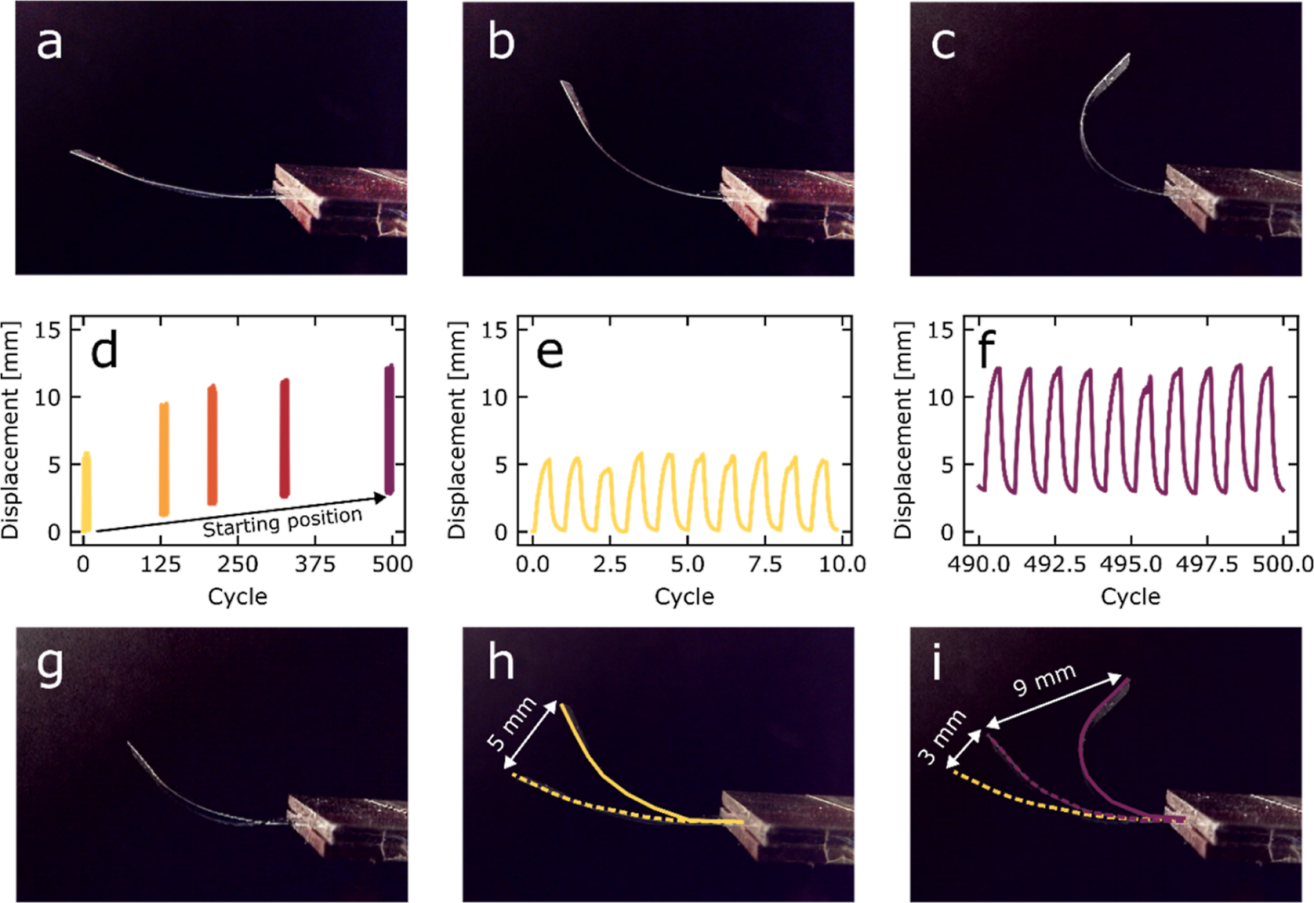
Fatigue test of an LCN with printed graphene tracks bending in response to heating caused by switching the potential “on” (14 V) and “off” (0 V) for intervals of 15 s each; five times ten cycles were captured on camera (cycles 1–10, 126–135, 204–213, 321–330, and 490–500). (a) Edge of the LCN actuator at the initial position before bending (at 0 V). (b) Displacement of the actuator at 14 V during the first cycle. (c) Displacement of the actuator at 14 V during cycle 500. (d) Displacement for captured bending cycles. (e) Expanded view of cycles 0–10. (f) Expanded view of cycles 490–500. (g) Displacement of the actuator at 0 V after actuating for 500 cycles (note that cycle starting positions, i.e., the initial bend, drift with cycle number as shown in panels (h,i). (h) Displacement of the edge of an LCN actuator during cycle 1 (yellow curve) with respect to the origin (yellow dashes). (i) Displacement during cycle 500 (purple curve) with respect to the starting position (yellow dashes) and to the cycle’s starting position (purple dashes).

We verified the bending of an actuator film clamped only on the end with the power connection upon reaching 60 °C. Upon application of 14 V, the LCN actuator bent with the planar aligned region on the inside of the curl, as expected (Figure 3a,b), and almost returned to its initial position after the potential was removed (Figure 3g).

Bending fatigue of an LCN actuator was tested by conducting 500 cycles of repeated switching “on” of the 14 V electrical potential for 15 s and switching “off” of the potential for 15 s. A camera was placed such that the actuator was seen edge-on, and a video of 10 consecutive cycles was captured approximately every 100 cycles from which the tip displacement was measured as a function of time, as is illustrated in Figure 3g–h. The actuator reliably performed all 500 cycles. No delamination was observed between the graphene heaters and the LCN substrates. A gradual shift in the tip’s starting position (i.e., initial bend) was observed from 0 mm during cycles 1–10 to approximately 3 mm during cycles 490–500 (Figure 3d). We hypothesize that the shift of the cycle starting position by the repetitive heating and bending at these relatively low temperatures is the result of the gradual release of internal stresses introduced during LCN photopolymerization (see also the Experimental Section). This introduction of internal stresses had already been observed previously and was attributed to the polymerization process in confinement between two glass substrates.^54^ We propose that this drift could be prevented by either preheating the sample or fully 3D printing the next generation of actuators, including the LCN substrates. This way, confinement between two glass plates and the corresponding internal stresses would be prevented.

The total film displacement increased from 5 mm during cycles 1–10 (Figure 3b,e,g) to 9 mm during cycles 490–500 (Figure 3c,f,h). The increase in displacement was accompanied by an increase in the average current passing through the track from 4 mA during the first ten cycles to 5–6 mA during the final ten cycles, even though the voltage was maintained constant at 14 V. This clearly indicates a decrease in resistance of the conductive track (*R* = *V*/*I*) resulting from repetitive bending leading to an increased power input and, thus, both increased temperature (*P* = *V*^2^/*R*) and bending displacement. For similar graphene tracks it was reported that after an initial increase, the resistance at 0% strain decreased by 57% after repetitive straining.^42^ This increase in conductivity is most likely a result of macroscopic rearrangements and degradation of the binder network in the track, causing rearrangement of junctions between the conductive graphene platelets. While graphene is abrasion resistant,^55,56^ the polymeric binder is not. Therefore, the polymeric binder is likely to already degrade at the relatively low bending strains in this study of 3–5% (see Figure S8 in the Supporting Information), which improves the contacts between the graphene platelets (i.e., decreases the interfacial resistance). Repetitive bending/straining therefore likely contributes to a decrease in the contact resistance. This observed decrease in resistance, in turn, increases the current passing through the material at a constant voltage. In addition, the temperature increase might induce thermal annealing, thereby further decreasing the resistance of the graphene heaters.

**Figure 4:**
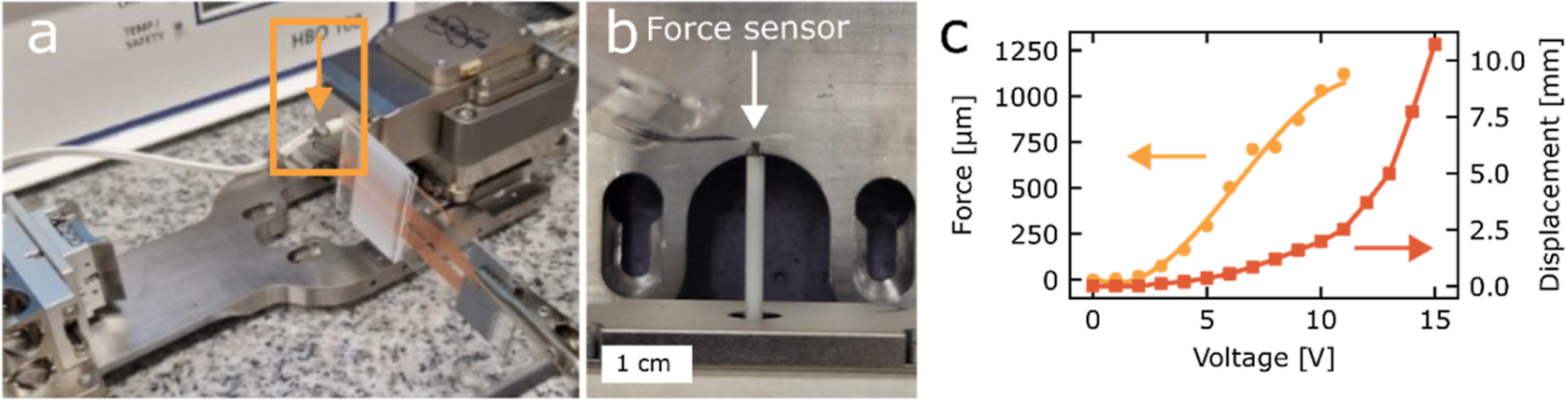
Bending force measurements. (a) Image of the setup used to determine bending forces. (b) Enlargement of the orange highlighted area in (a) displaying the Hysitron force sensor (indicated with arrows in both panels (a,b) with an LCN-graphene actuator pressing on it. (c) Force (orange dots) and displacement (red squares) versus voltage. The offset in bending force between 7 and 8 V was caused by having to readjust the zero position. The solid curve connecting the force measurements was obtained as a third-order polynomial fit to the data. Corresponding bending angle and temperature data are provided in Figure S8.

In addition to bending fatigue, the achievable bending force is a key parameter of actuators for applications. We gained first insights by measuring the force generated by our actuators by positioning the tip against a force sensor upon application of potentials of up to 11 V (corresponding to 50 °C and 2.5 mm displacement; see the setup in Figure 4a,b). This experiment showed that a maximum force of 1100 μN could be generated (Figure 4c). Larger forces may be achievable at higher temperatures, i.e., higher potentials (Figures S7–S8), which could unfortunately not be tested due to the sample origin disconnecting from the force sensor. While the measured force here seems low compared to devices described in the literature capable of lifting objects,^57^ the experimental conditions make a direct comparison difficult as we used a bending setup rather than weightlifting. An advantage of our measurement setup is its variable configuration and accurately calibrated force measurements with a more reproducible setup for higher voltages adapted to the LC actuators being developed.

**Figure 5:**
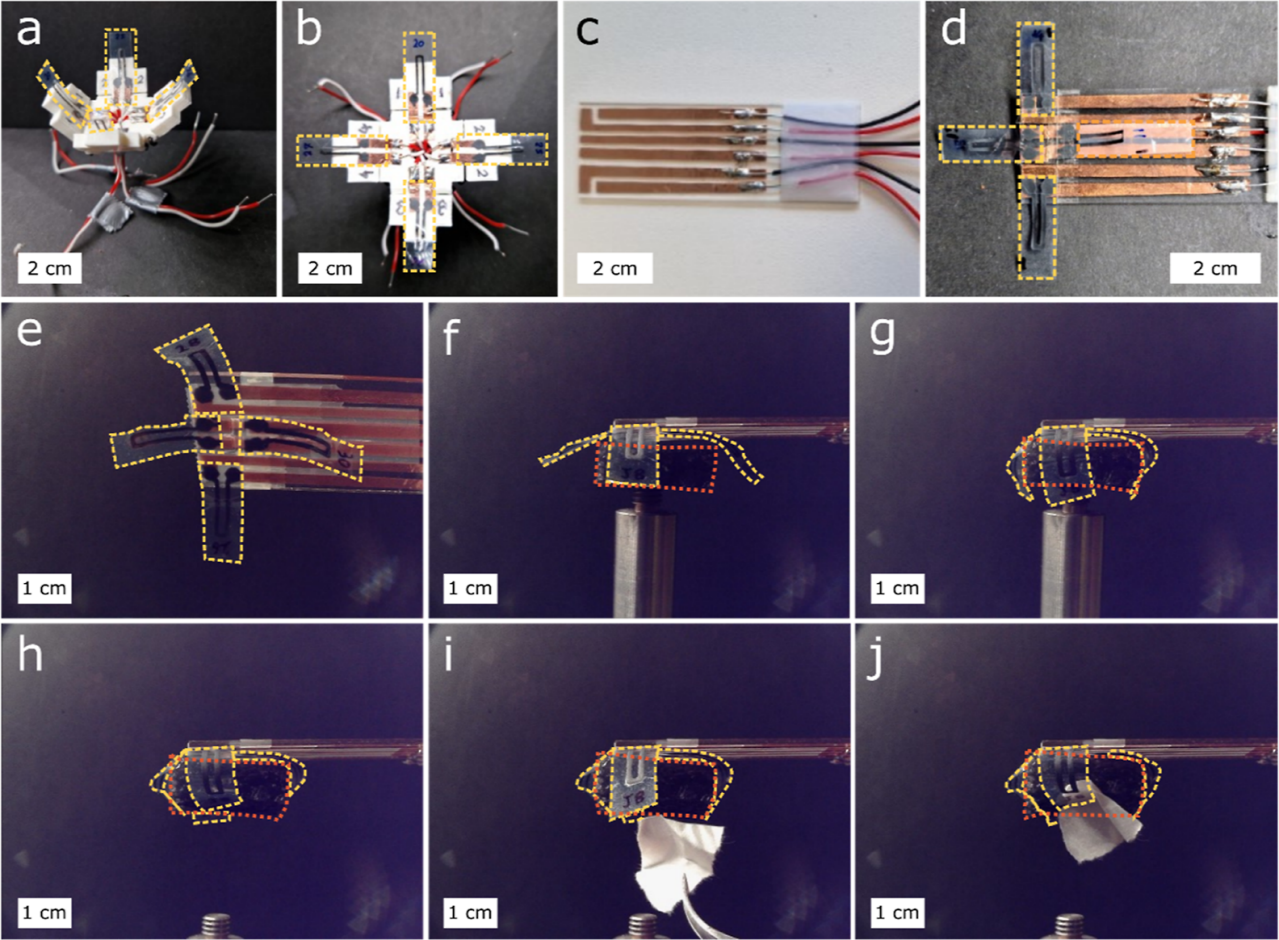
Two prototype gripper devices. (a,b) 3D-printed hinged holder with four graphene printed LCN strips (highlighted with yellow dashes) photographed from the (a) side and (b) top. (c–j) Flat gripper device. (c–d) Copper tape connection and wiring of the flat gripper (c) without and (d) with four graphene printed LCN fingers connected to copper tape strips. (e–j) Captured video stills of the flat prototype performing complex gripping via independent activation of the devices fingers: (e) bottom view of the device without the object showing the actuator assembly, (f) initial position before gripping the object (highlighted with red dots) on top of a metallic support, (g) gripping the object with all four fingers activated, (h) lifting the object away from the support, (i) individual release of one finger to allow gripping of a second object (a strip of paper), and (j) gripping of the second object. The object’s weight is approximately 70–100 mg.

Having established the concept of individually addressable actuators based on printing graphene on an LCN, demonstrating fully functioning single “fingers” and testing the ability to actuate individual fingers via an electrical signal (Video S1), several prototype gripper devices were created, of which the two most promising are presented in Figures 5 and S9 and in Videos S2, S3 and S4. Each device consists of two sets of opposing actuators for optimal gripping efficiency. The first device uses a 3D-printed hinged holder (Figure 5a,b), and the second is a flat sample holder made of a 3 × 8 cm^2^ glass slide (Figure 5c,d). Copper tape strips make electrical contact between the actuators and the wires connected to the control breadboard, which contains on/off switches for each individual finger. The devices were operated at a current of ca 10 mA and a voltage of 18–20 V. Their functioning was tested by lifting polystyrene objects weighing 70–100 mg and independently moving each LCN “finger” of the device (also see Videos S2 and S3 in the Supporting Information). The actuator response to the electric current is almost instantaneous (Video S1); we estimate that it is on the same order of magnitude as photoinduced responses.^58^

A subsequent experiment demonstrated the versatility of the gripper (Figure 5e–j). After gripping an object resting on a metallic stand and lifting it clear of the support, one of the fingers was opened by reducing the applied current, and a slip of paper was inserted between the finger and the object. The finger was reactivated and, upon closing, gripped the paper slip as well, after which it may be held indefinitely until the power is switched off, cooling and hence opening the fingers (also see Video S4). This shows that by being able to individually address each “finger” of the device, complex gripping actions similar to those of a human hand can be realized.

This work paves the way for all-3D-printed, all-organic, and thermally responsive LCN actuators. By avoiding the use of metal wiring or other cumbersome constructs, the possibility of printing multilayer actuators with various alignments and variously configured graphene tracks will dramatically expand the range of allowable actuator motions in the future.

## 3 Conclusions

Here we have used printed graphene-based heating elements to thermally drive the actuation of two gripper devices consisting of four individually addressable LCN actuators. Each of the four fingers of the device was made of splay-aligned LCNs. Graphene-based conductive tracks were successfully deposited on top of the LCN actuators by using a DIW printing method, which to the best of our knowledge has not been done before. The graphene tracks function as resistive heaters upon application of an electrical current/voltage. The heat is then transferred to the LCNs via their interface with the heater, causing the LCN actuator to bend and ultimately dissipate via their interfaces to the environment. Each finger of the gripper device may be addressed individually using a breadboard with “on”/”off” switches connected to each of the four resistive graphene heaters. A heat transfer model was established, validated, and aids rational device design by providing good estimates of dimensions, power production, and necessary resistance required to reach the desired temperature for actuation while maintaining safe electrical potentials. Regarding actuation, the LCN films were shown to reach an 11 mm bending magnitude (displacement) and a bending force of 1.10 mN upon applying 0–15 V, while the tracks can reach temperatures of up to 70 °C with 0–16 V input. The LCNs bending strain was approximately 3–5%, and the durability of the LCN finger was confirmed by repetitive on-and-off switching for 500 cycles. Notably, there was no indication of fatigue but rather an improvement to the bending range of the LCN caused by improved conductivity of the graphene track through repeated bending.

Finally, the two most promising gripper prototypes were presented demonstrating the device’s ability to grip and lift objects of modest masses (70–100 mg) and perform complex gripping by individually controlling one of the device’s fingers to grip and lift an additional object, similar to an actual hand. The design guidance offered by the heat transfer model and the flexibility of the LC-based system, which could be easily extended to LCNs with different actuation temperatures and rigidity levels or other modes of action such as twisting or spiraling by changing the LC alignment, offers a toolbox for the development of a wide range of all-organic heat-actuated soft robotics devices. The combination of durable and corrosion-resistant materials and selective actuation of individual fingers of the devices moves the field of soft robots capable of carrying out complex gripping actions one step further toward real-life applications.

## 4 Experimental Section

### 4.1 Materials

LC monomers 4-methoxyphenyl-4-(6-acryloyloxyhexyloxy)benzoate (RM105) and 1,4-di(4-(6-acryloyloxyhexyloxy)benzoyloxy)-2-methylbenzene (RM82) were obtained from Merck. Photoinitiator bis(2,4,6-trimethylbenzoyl)phenyl phosphine oxide (Irgacure 819) was obtained from Ciba (see Figure S1 for the chemical structures). Dichloromethane (DCM), ethyl acetate (EtAc) (both Biosolve Chimie), isopropyl alcohol (IPA, VWR Chemical), sulfuric acid (95–97%, Merck KGaA), ethyl cellulose (22 cP, Sigma-Aldrich), potassium permanganate (Sigma-Aldrich), propylene glycol ether (Sigma-Aldrich), and NeoRez-U431 binder (Covestro), were used as received.

### 4.2 Preparation of LC Actuators

The LCNs were produced by utilizing a published protocol.^46^ Briefly, RM105 and RM82 were combined in a 1:1 weight ratio in a glass vial, and 1 wt % of the photoinitiator Irgacure 819 was added. DCM was used as a solvent to improve the mixing of the monomers. The mixture was stirred on a hot plate at 80 °C for 30–60 min. The vial was then uncapped and placed on an 80 °C hot plate for 24–36 h to evaporate the DCM.

Clean 3 × 3 cm^2^ borosilicate glass slides were coated with either an Optmer AL1254 by JSR Corp for planar alignment or a Sunever 5661 by Nissan Chemical Corp for homeotropic alignment. The polyimides were spin coated on the glass plates for 5 s at 500 rpm followed by 45 s at 5000 rpm. The coated slides were then prebaked for 10–30 min at 90 °C on a hot plate and then baked for 90 min at 180 °C in an oven. After baking, the glass slides were allowed to cool, and the planar glass slides were rubbed on a velvet cloth. Scotch tape of approximately 50 μm thickness was placed along the edges of the planar glass slides parallel to the alignment/rubbing direction to function as spacers. One glass plate with planar alignment and one with homeotropic alignment were affixed to each other using a UV glue, resulting in an LC splay-alignment cell.

The cells were filled on a 90 °C hot plate with the LC mixture in the isotropic phase through capillary action. Filled cells were cooled to 55 °C (the nematic phase), and photopolymerization was initiated by exposing the sample for 180 s to UV light from an EXFO Omnicure S2000 (dose ∼3–5 mW cm^–2^, wavelengths 320–500 nm). The exposed cell was then heated for 30 min on a 120 °C hot plate to ensure the polymerization had reached maximum conversion. The cells were then opened by inserting a razor blade at the edge of the cell and carefully twisting the cell at the glue locations. Only one of the glass slides was removed prior to printing to ensure a flat substrate. When both glass slides were removed, the sample would curl up due to internal stresses induced during polymerization in the confinement of the two glass cells.^54^ The sample could be flattened by heating to 120 °C for a few cycles of 10 min.

### 4.3 Preparation of Graphene Ink

The graphene ink was prepared from thermally expanded graphite (TEG) following a method published previously.^39,42^ Briefly, 0.75 g of ethyl cellulose was dispersed in 500 mL of ethyl acetate in a 1 L glass cylinder, followed by adding 5 g of TEG and exfoliating for 90 min at 7000 rpm with an Ystral X40/38 high-shear mixer equipped with a 35 mm stator and a 25 mm rotor while cooling on ice. 22.5 g of NeoRez-U431 polyurethane-based binder was then added directly to the glass cylinder and mixed at 5000 rpm for 5 min, followed by the addition of 45 g of propylene glycol ether and mixing for 10 min. The mixture was transferred to a round-bottomed flask and placed on a Hei-VAP precision rotavapor. The volatile exfoliation solvent (ethyl acetate) was evaporated at 73 °C and 200 mbar. The ink was stored in plastic syringes until it was used for printing.

A Hyrel3D ENGINE HR printer equipped with a KR2 printhead (nozzle size 686 μm) was used for DIW. The printing patterns were created using Inkscape software and converted to G-code using the Gcodetools plug-in. The following printing parameters were used: 400 mm/min printing speed, 0.5 flow multiplier, 1350 pulses μL^–1^, and a height of 0.608 mm. The graphene tracks were applied to the homeotropic LCN alignment surface. The LCN sample with the printed graphene track was separated from the planar-aligned LC glass slide by carefully inserting a razor blade between the LCN and the glass slides and lifting the film off. This way, 35 graphene track samples were printed on LCNs, which were then cut to 15 mm × 5 mm strips.

### 4.4 Sample Analysis

Printed graphene track heights were analyzed with a Veeko DektakXT profilometer. Temperature profiles were imaged by using a Fluke Ti32 IR camera. Resistance values were obtained with a Voltcraft VC840 multimeter. DSC measurements were conducted with a TA Instruments DSC Q2000 using three heating and two cooling ramps with rates of 10 and 5 °C min^–1^, respectively. After the cooling ramp, the sample was left at 0 °C for 30 min. DMTA was performed on a planar aligned sample (9.1 × 5.10 × 0.122 mm^3^) along the alignment direction using a TA Instruments Q800 apparatus in vertical tension mode. Thermographs were obtained between 25 and 120 °C at a heating rate of 5 °C min^–1^ with 0.01 N preload force, 10 μm amplitude, and a 1 Hz oscillating frequency.

Bending force measurements were performed using a Bruker Hysitron PI-89 SEM PicoIndenter in a low load configuration without an indenter tip. Images of the fatigue tests (movie frames) and the bending force measurements were analyzed using Tracker software to quantify bending displacements and bending angles (see Figure S8).^59^

## Data Availability

All data and additional videos relevant for reproducing the findings in this manuscript are accessible via the 4TU ResearchData repository (https://data.4tu.nl/datasets/703fd633-9de8-4977-a05f-4b648cb1e94d).^52^

## Supporting Information Available

Liquid crystal network materials; differential scanning calorimetry; polarized optical microscopy of the LC mix; dynamic mechanical analysis of the liquid crystal network; heat transfer model; profilometry of printed tracks; bending force measurements; bend angle and bending strain estimation; and gripper hinged prototype (PDF)
Fully functioning single “fingers” and the ability to actuate individual fingers via an electrical signal (MP4)
Prototype gripper device 1 (MP4)
Prototype gripper device 2 (MP4)
Prototype gripper device 2—complex gripping of multiple objects (MP4)
