# Supplementary material for: Hot Fingers: Individually Addressable Graphene-Heater Actuated Liquid Crystal Grippers
Source: ACS Appl Mater Interfaces. 2024 Jun 13;16(25):32739–47. doi: 10.1021/acsami.4c06130 (PMC11212024; doi:10.1021/acsami.4c06130)
Supplement: Supplementary file 1 — am4c06130_si_001.pdf [file am4c06130_si_001.pdf]

Supporting Information

Hot Fingers: Individually Addressable Graphene-Heater Actuated Liquid Crystal Grippers

Laura S. van Hazendonk[1,2,±], Zafeiris J. Khalil[1,±], Wilko van Grondelle[1], Levina E. A. Wijkhuijs[1,2], Ingeborg Schreur-Piet[1,3], Michael G. Debije[2,4,], Heiner Friedrich[1,2,3,*]*

[1] Laboratory of Physical Chemistry, Department of Chemical Engineering and Chemistry Eindhoven University of Technology, PO box 513, Eindhoven, MB, 5600 The Netherlands

[2] Institute for Complex Molecular Systems, Eindhoven University of Technology, PO box 513, Eindhoven, MB, 5600 The Netherlands

[3] Center for Multiscale Electron Microscopy, Department of Chemical Engineering and Chemistry, Eindhoven University of Technology, PO box 513, Eindhoven, MB, 5600 The Netherlands

[4] Stimuli-responsive Functional Materials and Devices (SFD), Department of Chemical Engineering and Chemistry, Eindhoven University of Technology, PO box 513, Eindhoven, MB, 5600 The Netherlands

[±]These authors contributed equally.

*email: H.Friedrich@tue.nl, M.G.Debije@tue.nl

Contents

S1. Liquid crystal network materials 2
S2. Differential Scanning Calorimetry 2
S3. Polarized Optical Microscopy of the LC mix 3
S4. Dynamic mechanical analysis of the liquid crystal network 4
S5. Heat transfer model 5
S6. Profilometry of printed tracks 6
S7. Bending force measurements 6
S8. Bend angle and bending strain estimation 7
S9. Gripper hinged prototype 8
S10. References 8

S1. Liquid crystal network materials

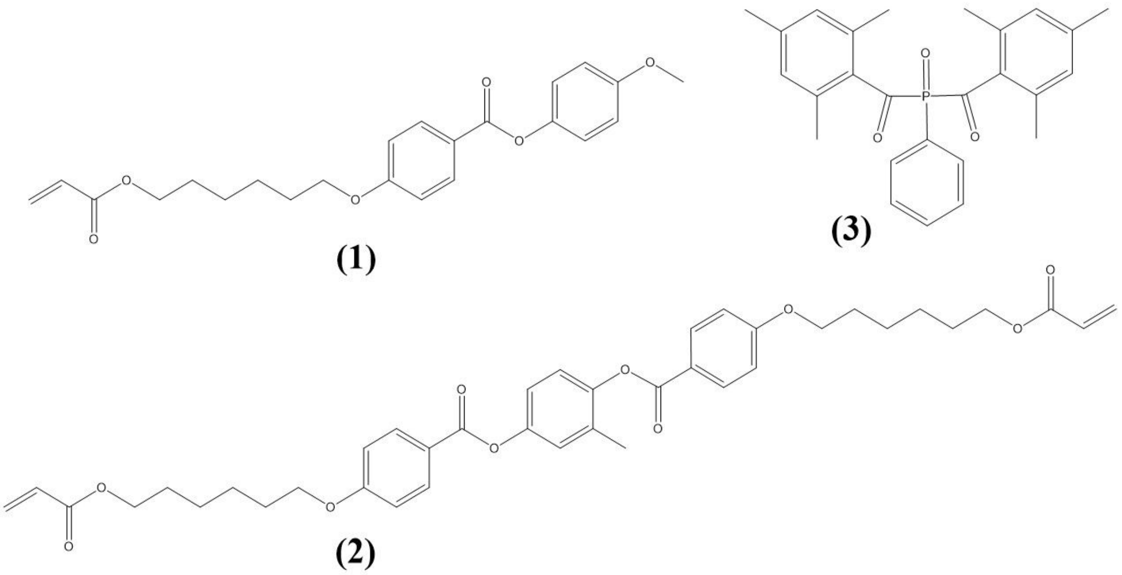

Figure S1. Materials used for the liquid crystal mixture (**1**) RM105, (**2**) RM82 and (**3**) Irgacure 819.

S2. Differential Scanning Calorimetry

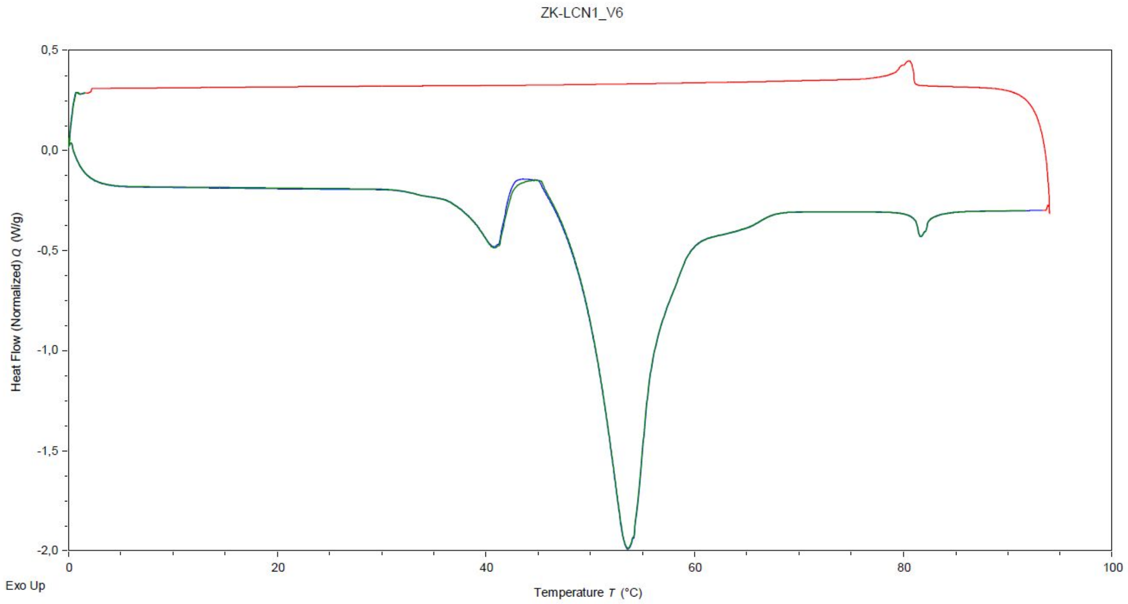

Figure S2: DSC heating and cooling ramps of the LCN mixture. The heating rate is 10 °C min^{-1} and cooling rate 5 °C min^{-1}. Three heat ramps and two cooling ramps were performed with a 30 min break in between cooling and heating. The first cycle of heating and cooling has been excluded from the plot.

S3. Polarized Optical Microscopy of the LC mix

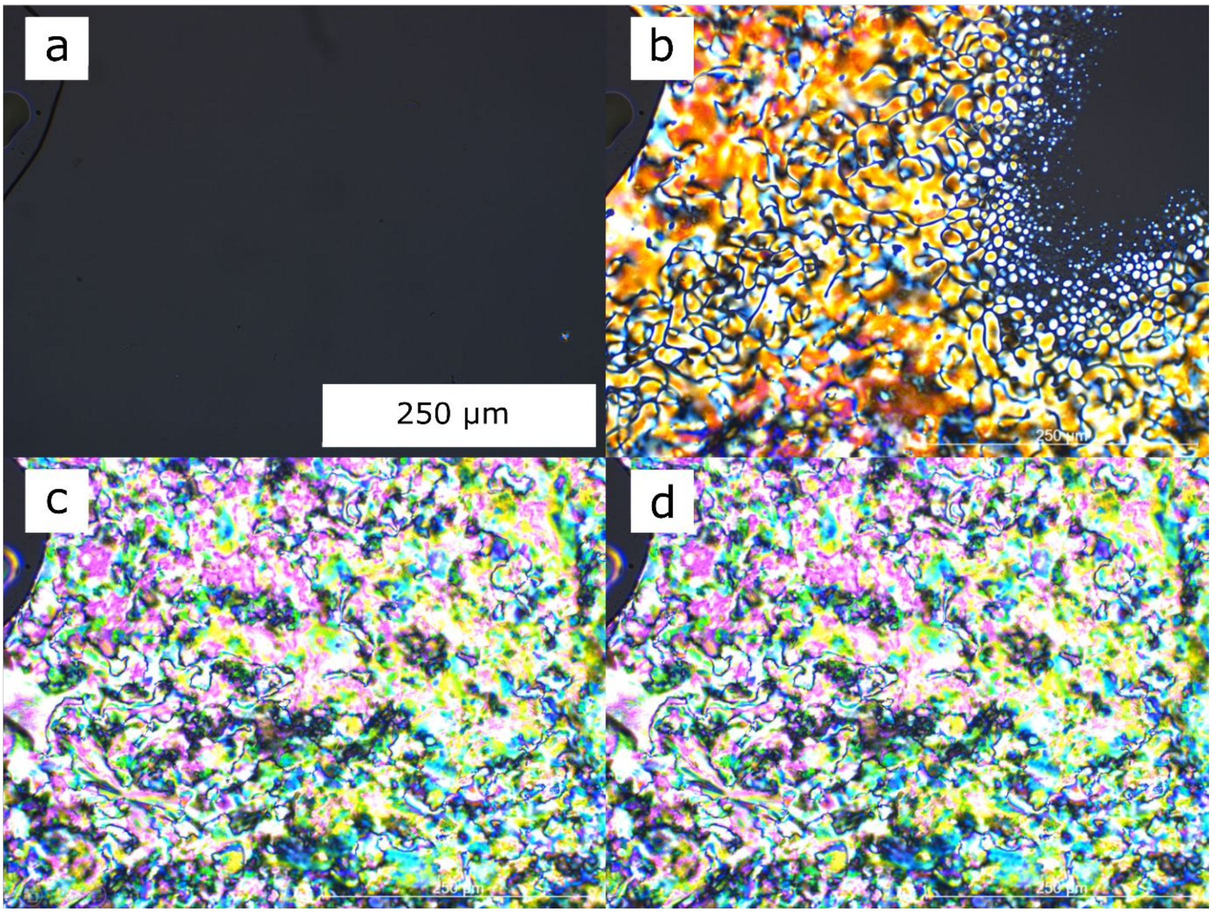

Figure S3: Optical microscopy images of the liquid crystal mixture in this work under crossed polarizers. a) After heating to 85 °C; b) after subsequent cooling to 82.5 °C; c) after further cooling to 55 °C; d) after 10 minutes holding at 55 °C. The scale bar in all panels is 250 µm.

The polarized optical microscopy (POM) results confirm that at temperatures beyond 85 °C (**Figure S3a)**, the LC mix is in the isotropic phase. In **Figure S3b**, the change in phase from isotropic to nematic is presented by the appearance of colorful domains. These domains indicate an increase in order in the LC mixture at 82.5 °C, which is in line with the DSC results in **Figure S2**. To emulate the method used to prepare the LCNs, specifically the step in which the glass cell filled with LC mix is cooled down to 55 °C, the sample was also imaged at 55 °C (**Figure S3c**). The image shows that the sample remained in the nematic phase after cooling, as no crystallization was observed. **Figure S3d** shows the sample region after ten minutes of holding at 55 °C. This confirms that the LC mix remained in the nematic phase for the full duration of the UV treatment/polymerization step (180 s).

S4. Dynamic mechanical thermal analysis of the liquid crystal network

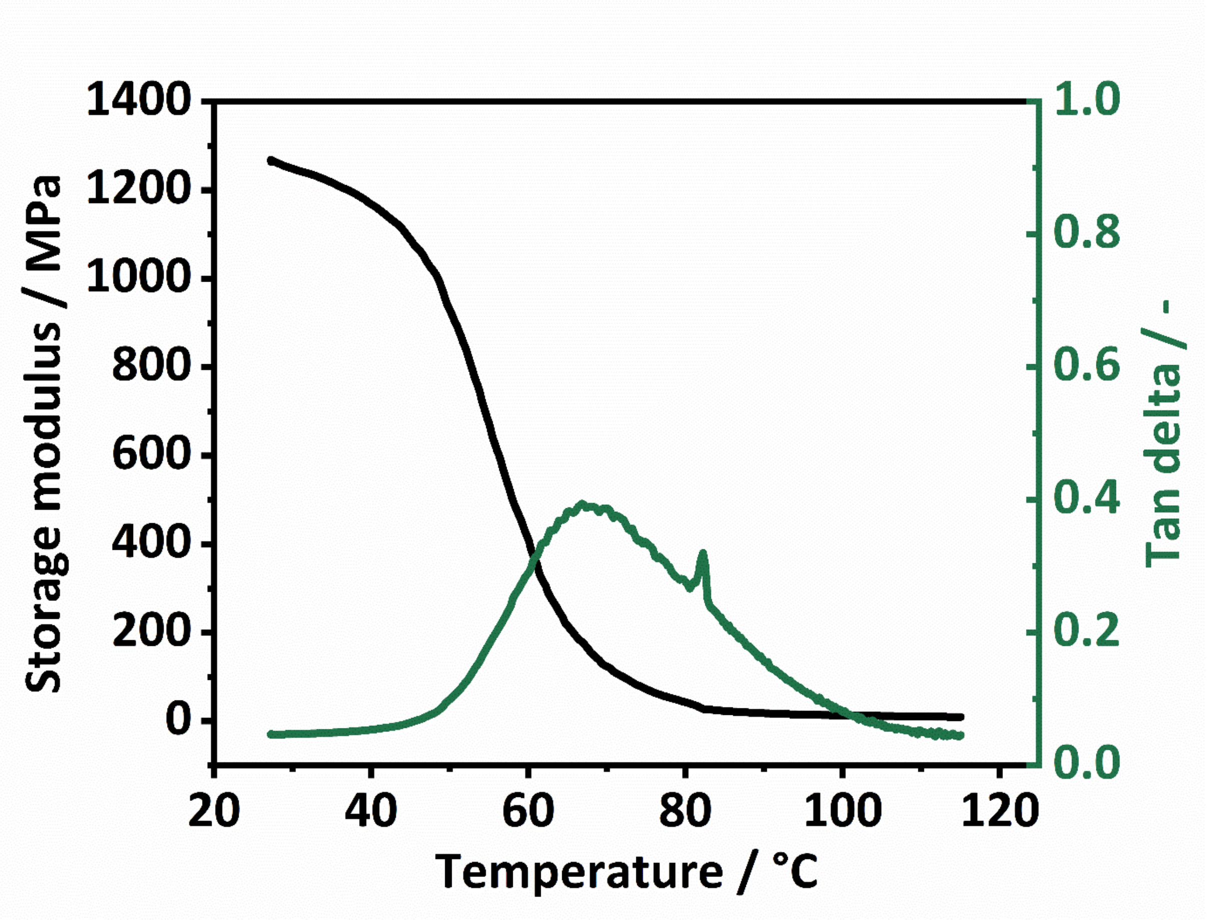

Figure S4: Dynamic mechanical thermal analysis (DMTA) of a planar LCN actuator along the alignment direction,with the storage modulus on the left axis (black) and Tan δ (loss tangent) on the right axis (green).

S5. Heat transfer model

The model input parameters are provided in Table S1, while the full model calculations are provided via the 4TU.ResearchData repository.[1]

Table S1. Model input parameters. The Excel sheet containing the full calculations is accessible via the 4TU.ResearchData repository.[1] Resistance values were obtained with a multimeter ($N = 27$), and track dimensions with profilometry ($N = 35$).

**Parameter**	**Unit**	**Value**
Nozzle diameter (D)	µm	686
Substrate thickness (t)	µm	50
Substrate width	mm	5
Substrate length	mm	15
Track length (L_p)	mm	26
Emissivity graphene (ε_G)	-	0.95
Emissivity LCN (ε_{LCN})	-	0.90
Stefan Boltzman constant (σ)	$W \cdot m^{-}2 \cdot K^{-4}$	$5.67 \cdot 10^{8}$
Air flow (v)	$m \cdot s^{-1}$	0.10
Resistance (R)	Ω	1957 ± 366
Track FWHM	µm	543 ± 174
Track height	µm	76 ± 17
Track base width	µm	891 ± 148

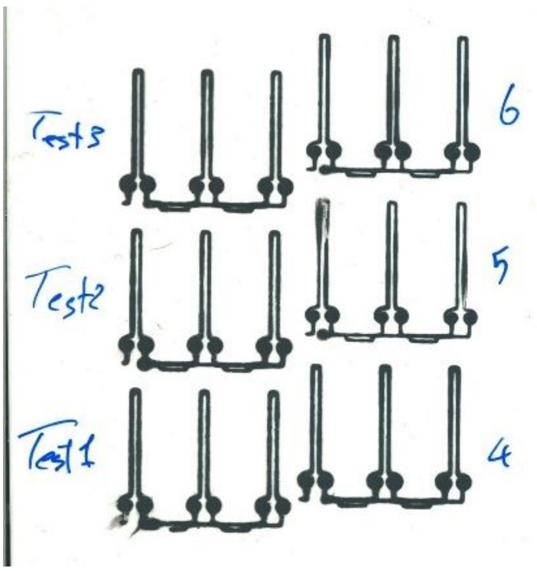

Figure S5: Initial trial prints on PET substrate used to get an estimate of the sample resistance for the heat model ($R \approx 2000$ Ω over a total path length of 26 mm).

S6. Profilometry of printed tracks

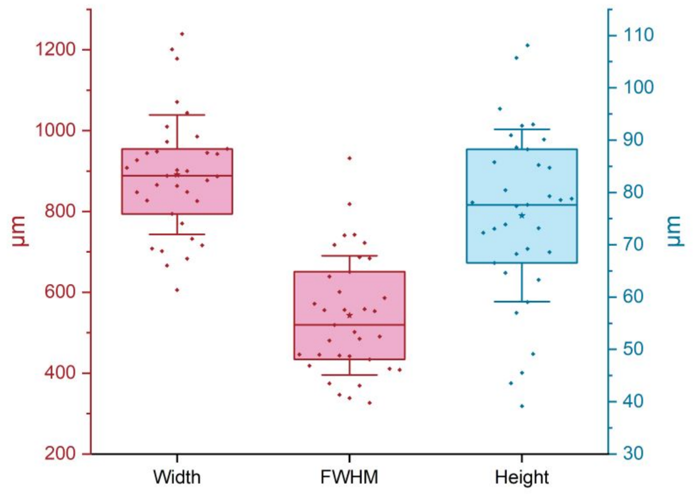

Figure S6: Box plots of base widths, FWHM (left axis) and heights (right axis) of 35 printed graphene tracks on top of LCN substrates characterized with profilometry.

S7. Bending force measurements

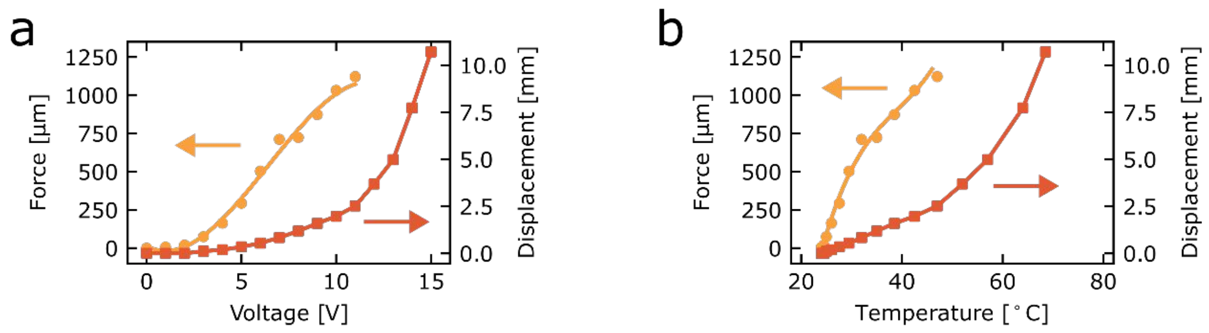

Figure S7. Force (orange dots) and displacement (red squares) versus voltage (a) and temperature (b).

S8. Bend angle and bending strain estimation

The bending strain ε was approximated as $\varepsilon = h/2r$,[2,3] with h the combined height of the substrate and the printed track and r the bending radius, which was measured with Tracker software as is shown in **Figure S8**. The bend angle could take on several values depending on the chosen bend radius due to the sample curvature varying along the substrate, most likely as a consequence of the graphene track not covering the full substrate length. This is illustrated in Figure **S8d-e,** where two different choices of bend radius (r = 2.0 vs r =1.3) for bending observed at 15 V yield bend angles of 142° versus 127°, giving strain levels of $\varepsilon_S \approx 0.032$ or $\varepsilon_S \approx 0.048$, respectively. It should be noted that the used strain equation might not be fully applicable to our situation as the printed layer was thicker than the substrate itself, nor did we account for the bilayer structure, which would require measuring the Young's moduli of both the tracks and the substrates.[2,3] Therefore, the reported strain levels should only be used as a rough approximation. The annotated dataset is provided via the 4TU.ResearchData repository.[1]

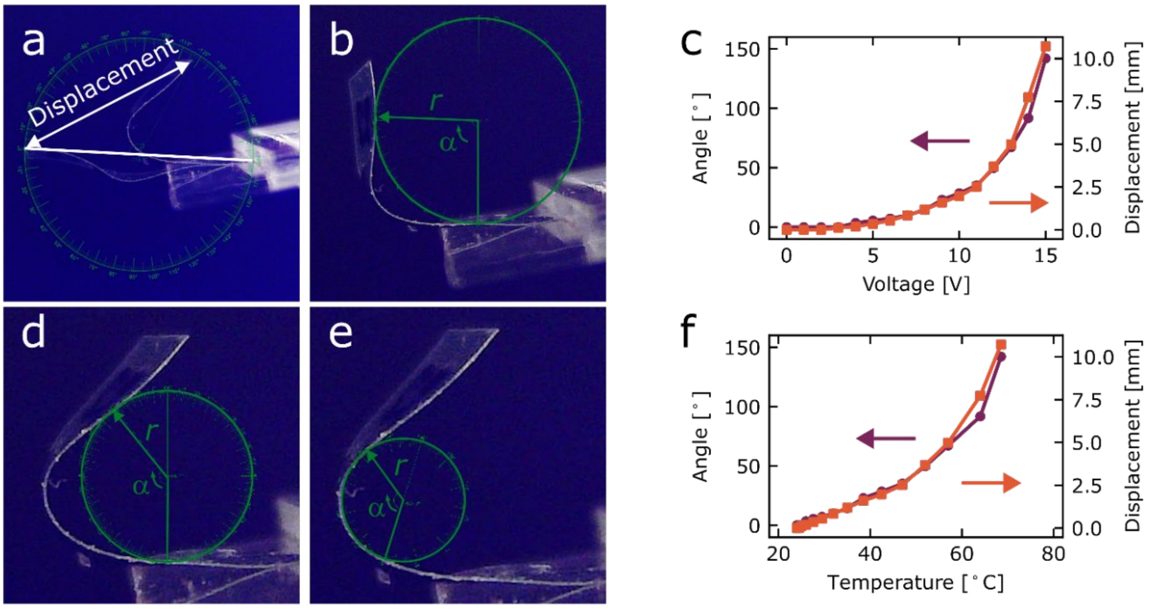

Figure S8. Displacement and bend angle measurements measured with Tracker software. a) Overlaid photographs of an LCN-G sample while flat and while bent with annotations of the substrate edge displacement with respect to the substrate origin before bending (0 V). b,d,e) Bend angle α and bend radius r (red squares) determination at 14 V (b) and 15 V (d, e). (d) and (e) illustrate two different choices of bend radius yielding different bend angles and hence different bending strain levels. c,f) Bend angle α and displacement versus voltage (c) and temperature (f), where at 15 V, the bend angle as determined in (b) and (d) is reported.

S9. Gripper hinged prototype

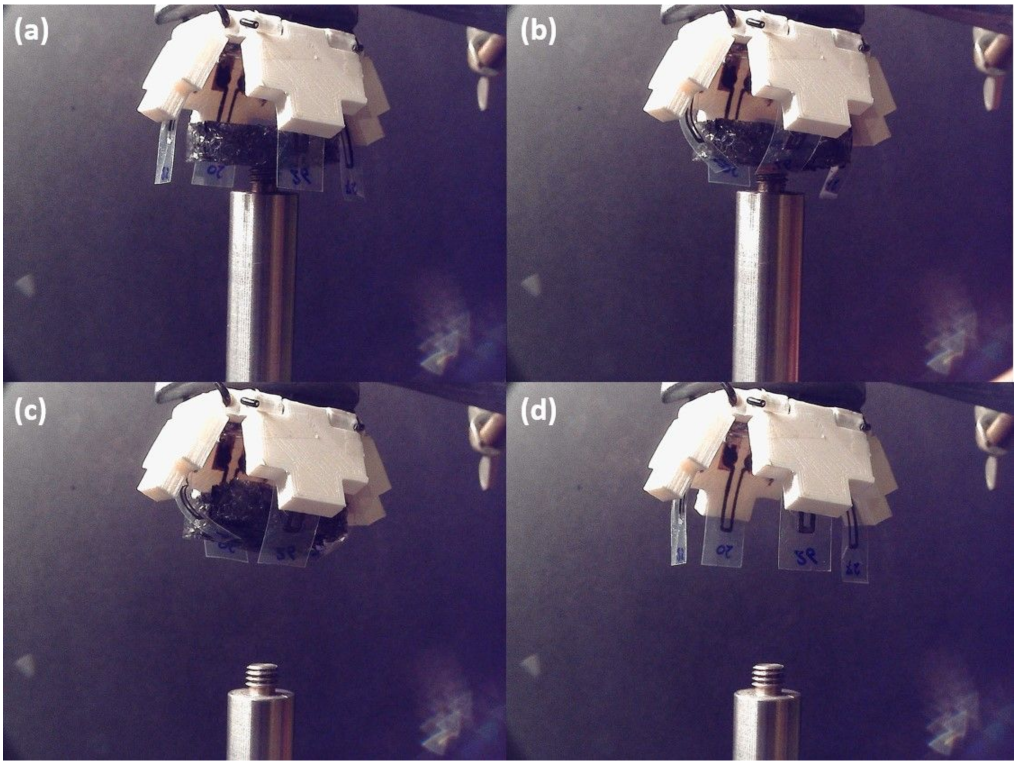

Figure S9 Photographs of the 3D printed hinged prototype: (a) before gripping, (b) after gripping the object, (c) while lifting the object, and (d) after releasing the object. The object weighs 70-100 mg.

S10. References

(1) Van Hazendonk, L. S.; Khalil, Z. J.; van Grondelle, W.; Wijkhuijs, L. E. A.; Schreur-Piet, I.; Debije, M. G.; Friedrich, H. Data Related to the Manuscript “Hot Fingers: Individually Addressable Graphene-Decorated Liquid Crystal Actuators,” 2023. https://doi.org/10.4121/cea38258-d81d-487e-a0e5-c3410a4225fa.

(2) Kim, T.-W.; Lee, J.-S.; Kim, Y.-C.; Joo, Y.-C.; Kim, B.-J. Bending Strain and Bending Fatigue Lifetime of Flexible Metal Electrodes on Polymer Substrates. *Materials* **2019**, *12* (15), 2490. https://doi.org/10.3390/ma12152490.

(3) Saleh, R.; Barth, M.; Eberhardt, W.; Zimmermann, A. Bending Setups for Reliability Investigation of Flexible Electronics. *Micromachines* **2021**, *12* (1), 78. https://doi.org/10.3390/mi12010078.
